# Supplementary material for: Resident Esophageal Microbiota Dysbiosis Correlates with Cancer Risk in Barrett’s Esophagus Patients and Is Linked to Low Adherence to WCRF/AICR Lifestyle Recommendations
Source: Nutrients. 2023 Jun 26;15(13):2885. doi: 10.3390/nu15132885 (PMC10343580; doi:10.3390/nu15132885)
Supplement: Supplementary file 1 [file nutrients-15-02885-s001.zip › nutrients-2451775-supplementary.pdf]

Table S1. Relative abundance values and statistical analyses of the bacteria phyla colonizing the distal esophagus in descending order of abundance in each group.

**Bacteria**	**Non-dysplastic BE**			**LGD**			**HGD**			**EAC**			**KW**	**Cuzick**	
	**median**	**Q1**	**Q3**	**median**	**Q1**	**Q3**	**median**	**Q1**	**Q3**	**median**	**Q1**	**Q3**	**p**	**p**	**z**
Firmicutes	41.00	29.00	48.00	39.00	28.25	41.75	34.00	26.00	45.50	41.00	39.00	54.00	ns	ns	/
Proteobacteria	22.00	13.00	41.00	30.00	23.25	46.25	26.00	14.50	43.00	19.00	17.00	20.50	ns	*	-2.54
Bacteroidetes	19.00	11.00	29.00	22.00	18.25	23.50	21.50	16.50	30.50	25.00	19.00	34.00	ns	ns	/
Actinobacteria	4.00	3.00	7.00	4.00	2.75	4.50	5.50	2.75	6.00	3.00	1.38	4.00	ns	ns	/
Fusobacteria	2.00	2.00	5.00	5.00	3.00	5.25	5.00	3.50	6.00	4.00	1.50	5.50	ns	ns	/
Epsilonbacteraeota	0.58	0.23	1.00	0.79	0.52	1.00	0.73	0.44	1.00	0.37	0.21	0.65	ns	ns	/
Patescibacteria	0.27	0.12	0.68	1.00	0.97	1.00	0.66	0.32	1.00	0.35	0.30	0.49	**	ns	/
Spirochaetes	0.04	0.00	0.13	0.11	0.06	0.19	0.02	0.00	0.09	0.03	0.03	0.05	ns	*	+2.17
Acidobacteria	0.01	0.00	0.04	0.00	0.00	0.00	0.00	0.00	0.02	0.00	0.00	0.00	ns	ns	/
Tenericutes	0.01	0.00	0.03	0.01	0.00	0.06	0.00	0.00	0.02	0.01	0.00	0.01	ns	ns	/
Synergistetes	0.00	0.00	0.03	0.04	0.03	0.07	0.00	0.00	0.03	0.00	0.00	0.01	ns	ns	/
Verrucomicrobia	0.00	0.00	0.00	0.00	0.00	0.02	0.00	0.00	0.00	0.00	0.00	0.00	ns	ns	/

Data are expressed as the median, first quartile (Q1), and third quartile (Q3). KW represents the p values resulted performing the Kruskal-Wallis test. Cuzick test for trend is expressed with the p value and, if significant, with the z value of trend. p values are annotated as *p<0.05,** p<0.01. ns represent a p value non-significant (p> 0.05). Non-dysplastic BE included patients with Barrett's esophagus; LGD included patients with low-grade dysplasia; HGD included patients with high-grade dysplasia; EAC included patients with esophageal adenocarcinoma.

Table S2. Relative abundance values and statistical analyses of the main bacteria classes (> 0.01%) colonizing the distal esophagus in descending order of abundance in each group.

Bacteria	Non-dysplastic BE			LGD			HGD			EAC			KW	Cuzick	
	median	Q1	Q3	median	Q1	Q3	median	Q1	Q3	median	Q1	Q3	p	p	z
Bacilli	31.00	17.00	41.00	26.00	23.00	30.75	20.00	18.50	31.50	30.00	24.50	31.50	ns	ns	/
Bacteroidia	19.00	11.00	29.00	22.00	18.25	23.50	21.50	16.50	30.50	25.00	19.00	34.00	ns	ns	/
Gammaproteobacteria	18.00	11.00	30.00	24.00	19.75	26.00	10.50	8.00	21.50	15.00	11.50	18.50	ns	ns	/
Negativicutes	6.00	2.00	9.00	6.00	4.25	6.25	8.00	3.00	10.50	11.00	5.00	17.00	ns	ns	/
Actinobacteria	4.00	2.00	6.00	4.00	2.00	4.25	4.50	2.75	5.25	3.00	1.35	3.50	ns	ns	/
Alphaproteobacteria	3.00	0.82	6.00	4.00	3.00	10.00	4.50	1.68	20.75	0.47	0.21	3.25	ns	ns	/
Fusobacteriia	2.00	2.00	5.00	5.00	3.00	5.25	5.00	3.50	6.00	4.00	1.50	5.50	ns	ns	/
Clostridia	1.00	1.00	3.00	2.00	1.75	3.25	2.00	1.70	2.75	2.00	1.00	4.50	ns	ns	/
Campylobacteria	0.58	0.23	1.00	0.79	0.52	1.00	0.73	0.44	1.00	0.37	0.21	0.65	ns	ns	/
Saccharimonadia	0.26	0.09	0.60	0.53	0.47	0.60	0.50	0.28	0.90	0.29	0.24	0.41	ns	ns	/
Coriobacteriia	0.23	0.05	0.66	0.20	0.05	0.38	0.40	0.09	0.55	0.49	0.08	0.52	ns	ns	/
Erysipelotrichia	0.16	0.05	0.29	0.28	0.22	0.36	0.29	0.11	0.52	0.23	0.12	0.54	ns	ns	/
Spirochaetia	0.04	0.00	0.13	0.11	0.06	0.19	0.02	0.00	0.09	0.03	0.03	0.05	ns	ns	/
Mollicutes	0.01	0.00	0.03	0.01	0.00	0.06	0.00	0.00	0.02	0.01	0.00	0.01	ns	ns	/

Data are expressed as the median, first quartile (Q1), and third quartile (Q3). KW represents the p values resulted performing the Kruskal-Wallis test. Cuzick test for trend is expressed with the p value and, if significant, with the z value of trend. p values are annotated as *p<0.05,** p<0.01. ns represent a p value non-significant (p> 0.05). Non-dysplastic BE included patients with Barrett's esophagus; LGD included patients with low-grade dysplasia; HGD included patients with high-grade dysplasia; EAC included patients with esophageal adenocarcinoma.

Table S3. Relative abundance values and statistical analyses of the main bacteria orders (> 0.01%) colonizing the distal esophagus in descending order of abundance in each group.

**Bacteria**	**Non-dysplastic BE**			**LGD**			**HGD**			**EAC**			**KW**	**Cuzick**	
	**median**	**Q1**	**Q3**	**median**	**Q1**	**Q3**	**median**	**Q1**	**Q3**	**median**	**Q1**	**Q3**	ns	ns	/
Lactobacillales	26.000	14.000	36.000	23.000	20.000	25.250	18.500	15.500	27.250	26.000	19.500	28.000	ns	ns	/
Bacteroidales	15.000	9.000	29.000	20.000	16.750	22.500	21.500	15.500	30.250	25.000	18.500	33.000	ns	*	+2.13
Pasteurellales	7.000	2.000	13.000	12.000	9.250	20.500	7.500	6.500	20.000	9.000	6.000	16.000	ns	ns	/
Selenomonadales	6.000	2.000	9.000	6.000	4.250	6.250	8.000	3.000	10.500	11.000	5.000	17.000	ns	ns	/
Betaproteobacteriales	3.000	0.820	6.000	2.000	0.844	5.250	1.394	0.728	3.000	3.000	1.000	4.500	ns	ns	/
Bacillales	2.000	1.000	4.000	3.000	1.713	4.500	2.000	2.000	3.000	4.000	2.500	6.000	ns	ns	/
Fusobacteriales	2.000	2.000	5.000	5.000	3.000	5.250	5.000	3.500	6.000	4.000	1.500	5.500	ns	ns	/
Micrococcales	2.000	0.659	3.000	2.000	0.874	2.000	2.500	1.660	3.000	1.000	0.650	1.500	ns	ns	/
Actinomycetales	1.000	0.412	2.000	1.000	0.478	1.000	1.500	0.782	2.250	0.720	0.391	0.962	ns	ns	/
Clostridiales	1.000	0.999	3.000	2.000	1.750	3.250	2.000	1.699	2.750	2.000	1.000	4.500	ns	ns	/
Sphingomonadales	0.963	0.116	3.000	4.000	0.674	9.500	3.500	0.888	15.750	0.243	0.101	2.234	ns	ns	/
Pseudomonadales	0.627	0.091	4.000	0.642	0.359	1.009	0.247	0.051	0.481	0.038	0.010	0.233	*	**	-2.69
Campylobacterales	0.576	0.231	1.000	0.789	0.518	1.000	0.730	0.441	1.000	0.373	0.208	0.654	ns	ns	/
Flavobacteriales	0.483	0.232	0.847	0.816	0.352	1.147	0.431	0.210	0.737	0.723	0.422	1.000	ns	ns	/
Saccharimonadales	0.262	0.087	0.601	0.528	0.467	0.595	0.502	0.278	0.904	0.289	0.237	0.415	ns	ns	/
Coriobacteriales	0.225	0.046	0.659	0.201	0.046	0.380	0.402	0.087	0.548	0.486	0.083	0.516	ns	ns	/
Erysipelotrichales	0.156	0.050	0.286	0.275	0.219	0.363	0.295	0.110	0.525	0.233	0.120	0.538	ns	ns	/
Corynebacteriales	0.126	0.039	0.266	0.121	0.043	0.434	0.061	0.027	0.116	0.025	0.019	0.078	ns	*	-2.19
Campylobacterales	0.576	0.231	1.000	0.789	0.518	1.000	0.730	0.441	1.000	0.373	0.208	0.654	ns	ns	/
Flavobacteriales	0.483	0.232	0.847	0.816	0.352	1.147	0.431	0.210	0.737	0.723	0.422	1.000	ns	ns	/
Saccharimonadales	0.262	0.087	0.601	0.528	0.467	0.595	0.502	0.278	0.904	0.289	0.237	0.415	ns	ns	/
Coriobacteriales	0.225	0.046	0.659	0.201	0.046	0.380	0.402	0.087	0.548	0.486	0.083	0.516	ns	ns	/
Bifidobacteriales	0.037	0.000	0.121	0.071	0.037	0.093	0.203	0.006	0.515	0.025	0.005	0.155	ns	ns	/
Spirochaetales	0.037	0.000	0.133	0.111	0.062	0.192	0.020	0.000	0.094	0.034	0.025	0.052	ns	ns	/
Acetobacterales	0.021	0.000	0.065	0.014	0.003	0.031	0.017	0.006	0.043	0.003	0.000	0.061	ns	ns	/
Cytophagales	0.015	0.000	0.048	0.014	0.000	0.034	0.016	0.000	0.033	0.000	0.000	0.032	ns	ns	/

Enterobacteriales	0.006	0.000	0.048	0.003	0.000	0.023	0.014	0.000	0.027	0.008	0.007	0.015	ns	ns	/

Data are expressed as the median, first quartile (Q1), and third quartile (Q3). KW represents the p values resulted performing the Kruskal-Wallis test. Cuzick test for trend is expressed with the p value and, if significant, with the z value of trend. p values are annotated as *p<0.05,** p<0.01. ns represent a p value non-significant (p> 0.05). Non-dysplastic BE included patients with Barrett's esophagus; LGD included patients with low-grade dysplasia; HGD included patients with high-grade dysplasia; EAC included patients with esophageal adenocarcinoma.

Table S4. Relative abundance values and statistical analyses of the main bacteria families (> 0.05%) colonizing the distal esophagus in descending order of abundance in each group.

**Bacteria**	**Non-dysplastic BE**			**LGD**			**HGD**			**EAC**			**KW**	**Cuzick**	
	**median**	**Q1**	**Q3**	**median**	**Q1**	**Q3**	**median**	**Q1**	**Q3**	**median**	**Q1**	**Q3**	**p**	**p**	**z**
Streptococcaceae	26.0000	13.0000	36.0000	22.0000	19.2500	24.2500	18.0000	14.7500	25.5000	24.0000	19.0000	26.0000	ns	ns	/
Prevotellaceae	14.0000	8.0000	24.0000	17.0000	10.7500	21.2500	19.5000	12.7500	27.2500	22.0000	13.5000	31.0000	ns	*	+2.08
Pasteurellaceae	7.0000	2.0000	13.0000	12.0000	9.2500	20.5000	7.5000	6.5000	20.0000	9.0000	6.0000	16.0000	ns	ns	/
Veillonellaceae	6.0000	2.0000	9.0000	6.0000	4.2500	6.2500	8.0000	3.0000	10.5000	11.0000	5.0000	17.0000	ns	ns	/
Family XI-01	2.0000	0.8250	4.0000	3.0000	1.6880	4.2500	2.0000	2.0000	3.0000	4.0000	2.5000	5.5000	ns	ns	/
Micrococcaceae	2.0000	0.5910	3.0000	2.0000	0.7788	2.0000	2.5000	1.6603	3.0000	1.0000	0.6500	1.5000	ns	ns	/
Porphyromonadaceae	2.0000	0.3130	3.0000	2.0000	2.0000	3.2500	2.5000	1.7500	3.0000	2.0000	0.4030	2.0000	ns	ns	/
Actinomycetaceae	1.0000	0.4120	2.0000	1.0000	0.4775	1.0000	1.5000	0.7820	2.2500	0.7200	0.3910	0.9615	ns	ns	/
Fusobacteriaceae	1.0000	0.6050	3.0000	2.0000	1.7500	3.0000	2.0000	1.0000	3.0000	2.0000	0.7875	4.5000	ns	ns	/
Leptotrichiaceae	1.0000	0.6170	3.0000	2.0000	1.0000	2.2500	2.5000	0.9253	3.5000	0.4790	0.3145	1.0000	ns	ns	/
Neisseriaceae	1.0000	0.4210	4.0000	2.0000	0.8145	4.2500	1.3750	0.5550	2.2500	3.0000	1.0000	4.0000	ns	ns	/
Sphingomonadaceae	0.9630	0.1160	3.0000	4.0000	0.6743	9.5000	3.5000	0.8875	15.7500	0.2430	0.1013	2.2340	ns	ns	/
Lachnospiraceae	0.6900	0.2700	2.0000	2.0000	0.5828	2.0000	2.0000	0.7303	2.5000	0.8740	0.4630	2.5000	ns	ns	/
Campylobacteraceae	0.5330	0.1880	1.0000	0.7890	0.5183	1.0000	0.7035	0.4410	1.0000	0.3730	0.2075	0.4755	ns	ns	/
Carnobacteriaceae	0.5140	0.2030	0.7600	0.8340	0.6303	1.0000	0.4840	0.3390	1.0000	0.7830	0.2025	0.9435	ns	ns	/
Moraxellaceae	0.4890	0.0563	4.0000	0.5970	0.3040	0.9695	0.1820	0.0464	0.4478	0.0152	0.0099	0.1935	*	**	-2.74
Beijerinckiaceae	0.2810	0.0548	0.8920	0.5340	0.2960	0.7030	0.2635	0.1317	1.2500	0.0380	0.0127	0.3904	ns	ns	/
Burkholderiaceae	0.2420	0.0889	0.5400	0.0742	0.0485	0.4870	0.0465	0.0420	0.1445	0.0195	0.0101	0.0648	**	**	-3.44
Weeksellaceae	0.2200	0.0818	0.4160	0.2680	0.1287	0.4855	0.1848	0.0753	0.3058	0.1530	0.0912	0.6060	ns	ns	/
Flavobacteriaceae	0.2030	0.0592	0.4330	0.3200	0.0245	0.4310	0.1860	0.1220	0.3520	0.1180	0.0613	0.5195	ns	ns	/

Atopobiaceae	0.1990	0.0450	0.6210	0.2010	0.0461	0.3595	0.3910	0.0865	0.5205	0.4650	0.0813	0.5115	ns	ns	/
Family XI-02	0.1930	0.0435	0.4390	0.3990	0.2515	0.4403	0.1200	0.1004	0.2445	0.0857	0.0738	0.1715	ns	ns	/
Saccharimonadaceae	0.1850	0.0467	0.5350	0.4450	0.3683	0.5335	0.3745	0.2280	0.9040	0.2050	0.1310	0.3275	ns	ns	/
Erysipelotrichaceae	0.1560	0.0495	0.2860	0.2750	0.2193	0.3630	0.2945	0.1104	0.5248	0.2330	0.1195	0.5375	ns	ns	/
Peptostreptococcaceae	0.1450	0.0378	0.4310	0.4390	0.3015	0.5263	0.0477	0.0065	0.0858	0.6370	0.1680	0.7440	**	ns	/
Ruminococcaceae	0.1050	0.0158	0.2060	0.1960	0.1238	0.2603	0.0850	0.0695	0.2425	0.0796	0.0721	0.0833	ns	ns	/
Family XIII	0.0932	0.0173	0.2070	0.2820	0.2318	0.3335	0.1253	0.0341	0.2603	0.0652	0.0463	0.1031	ns	ns	/
Corynebacteriaceae	0.0818	0.0190	0.1830	0.0549	0.0255	0.2708	0.0530	0.0086	0.0708	0.0245	0.0193	0.0666	ns	ns	/
Caulobacteraceae	0.0743	0.0138	0.3450	0.2090	0.1023	0.2503	0.2845	0.0223	0.4433	0.0123	0.0031	0.1354	ns	ns	/
Pseudomonadaceae	0.0669	0.0126	0.2140	0.0600	0.0436	0.1051	0.0240	0.0051	0.0534	0.0163	0.0000	0.0326	ns	*	-2.39
Xanthomonadaceae	0.0606	0.0243	0.2020	0.1020	0.0391	0.3163	0.0131	0.0033	0.0276	0.0040	0.0000	0.1592	ns	*	-2-28
Staphylococcaceae	0.0590	0.0296	0.1480	0.1280	0.0558	0.1568	0.0472	0.0232	0.0854	0.0099	0.0045	0.0472	ns	ns	/
Rhizobiaceae	0.0532	0.0089	0.1100	0.0485	0.0090	0.0609	0.0253	0.0091	0.0353	0.0000	0.0000	0.0208	*	**	-2.77

Data are expressed as the median, first quartile (Q1), and third quartile (Q3). KW represents the p values resulted performing the Kruskal-Wallis test. Cuzick test for trend is expressed with the p value and, if significant, with the z value of trend. p values are annotated as *p<0.05,** p<0.01. n.s. represent a p value non-significant (p> 0.05). Non-dysplastic BE included patients with Barrett's esophagus; LGD included patients with low-grade dysplasia; HGD included patients with high-grade dysplasia; EAC included patients with esophageal adenocarcinoma.

Table S5. Relative abundance values and statistical analyses of the main bacteria genera (> 0.05%) colonizing the distal esophagus in descending order of abundance in each group.

**Bacteria**	**Non-dysplastic BE**			**LGD**			**HGD**			**EAC**			**KW**	**Cuzick**	
	**median**	**Q1**	**Q3**	**median**	**Q1**	**Q3**	**median**	**Q1**	**Q3**	**median**	**Q1**	**Q3**	**p**	**p**	**z**
Streptococcus	26.000	13.000	36.000	22.000	19.250	24.250	18.000	14.750	25.500	24.000	19.000	26.000	ns	ns	/
Prevotella	9.309	6.236	17.576	12.827	7.227	14.381	15.820	7.746	20.509	19.349	10.193	21.903	ns	ns	/
Haemophilus	6.000	2.000	10.000	8.000	4.685	12.750	5.000	4.250	11.500	7.000	2.500	14.500	ns	ns	/
Veillonella	5.000	2.000	7.000	4.000	3.500	5.250	6.500	3.000	9.250	10.000	4.500	16.500	ns	ns	/
Alloprevotella	3.000	0.625	5.000	5.000	3.750	5.250	3.500	3.000	6.250	3.000	2.500	7.500	ns	*	+2.03
Gemella	2.000	0.825	4.000	3.000	1.688	4.250	2.000	2.000	3.000	4.000	2.500	5.500	ns	ns	/
Porphyromonas	2.000	0.313	3.000	2.000	2.000	3.250	2.500	1.750	3.000	2.000	0.403	2.000	ns	ns	/
Rothia	2.000	0.549	3.000	1.000	0.639	2.000	2.500	1.657	3.000	1.000	0.640	1.500	ns	ns	/

Actinomyces	1.000	0.412	2.000	1.000	0.476	1.000	1.500	0.781	2.250	0.716	0.381	0.962	ns	ns	/
Fusobacterium	1.000	0.605	3.000	2.000	1.750	3.000	2.000	1.000	3.000	2.000	0.788	4.500	ns	ns	/
Leptotrichia	1.000	0.513	2.000	2.000	0.932	2.000	2.500	0.925	3.500	0.479	0.258	1.000	ns	ns	/
Neisseria	0.910	0.278	4.000	2.000	0.791	3.750	1.360	0.343	2.250	2.000	0.940	4.000	ns	ns	/
Sphingomonas	0.884	0.113	3.000	4.000	0.580	9.250	3.500	0.856	15.000	0.236	0.067	2.234	ns	ns	/
Campylobacter	0.533	0.188	1.000	0.789	0.518	1.000	0.704	0.441	1.000	0.373	0.208	0.476	ns	ns	/
Acinetobacter	0.486	0.051	4.000	0.463	0.219	0.717	0.120	0.039	0.448	0.015	0.010	0.187	ns	**	-2.71
Granulicatella	0.465	0.186	0.760	0.834	0.610	1.000	0.470	0.329	1.000	0.783	0.201	0.942	ns	ns	/
Methylobacterium	0.273	0.050	0.719	0.317	0.278	0.455	0.264	0.122	1.250	0.030	0.011	0.369	ns	ns	/
Megasphaera	0.271	0.067	1.000	0.419	0.093	0.599	0.868	0.114	1.000	0.142	0.093	0.600	ns	ns	/
Atopobium	0.199	0.045	0.562	0.201	0.046	0.334	0.365	0.085	0.510	0.459	0.078	0.504	ns	ns	/
Actinobacillus	0.186	0.011	2.000	2.000	0.757	2.250	0.160	0.082	0.481	1.000	0.539	1.000	ns	ns	/
Oribacterium	0.183	0.051	0.398	0.371	0.132	0.406	0.317	0.086	0.540	0.123	0.086	0.200	ns	ns	/
Lachnoanaerobaculum	0.177	0.060	0.436	0.179	0.123	0.280	0.299	0.096	0.703	0.160	0.085	1.277	ns	ns	/
Capnocytophaga	0.158	0.032	0.409	0.314	0.025	0.415	0.183	0.120	0.352	0.118	0.054	0.501	ns	ns	/
Stomatobaculum	0.134	0.028	0.251	0.100	0.076	0.214	0.253	0.108	0.536	0.245	0.094	0.698	ns	ns	/
TM7 phylum	0.134	0.027	0.386	0.260	0.151	0.513	0.325	0.079	0.645	0.109	0.078	0.257	ns	ns	/
Selenomonas	0.111	0.060	0.216	0.182	0.071	0.264	0.288	0.066	0.316	0.084	0.059	0.106	ns	ns	/
Parvimonas	0.100	0.007	0.278	0.285	0.224	0.428	0.111	0.091	0.215	0.080	0.043	0.157	ns	ns	/
Peptostreptococcus	0.077	0.005	0.198	0.249	0.211	0.342	0.039	0.007	0.058	0.532	0.137	0.661	**	ns	/
Brevundimonas	0.067	0.011	0.262	0.137	0.071	0.219	0.282	0.022	0.441	0.012	0.003	0.135	ns	ns	/
Pseudomonas	0.067	0.013	0.214	0.060	0.044	0.105	0.024	0.005	0.053	0.016	0.000	0.033	ns	*	-2.45
Bergeyella	0.063	0.000	0.247	0.195	0.114	0.480	0.143	0.050	0.294	0.142	0.056	0.601	ns	ns	/
Aggregatibacter	0.060	0.006	0.220	0.253	0.088	1.378	2.000	1.000	3.000	0.122	0.038	0.164	***	**	+2.66
Staphylococcus	0.050	0.029	0.145	0.128	0.056	0.155	0.047	0.021	0.085	0.008	0.004	0.046	ns	ns	/

Data are expressed as the median, first quartile (Q1), and third quartile (Q3). KW represents the p values resulted performing the Kruskal-Wallis test. Cuzick test for trend is expressed with the p value and, if significant, with the z value of trend. p values are annotated as *p<0.05,** p<0.01. ns represent a p value non-significant (p> 0.05). Non-dysplastic BE included patients with Barrett's esophagus; LGD included patients with low-grade dysplasia; HGD included patients with high-grade dysplasia; EAC included patients with esophageal adenocarcinoma.

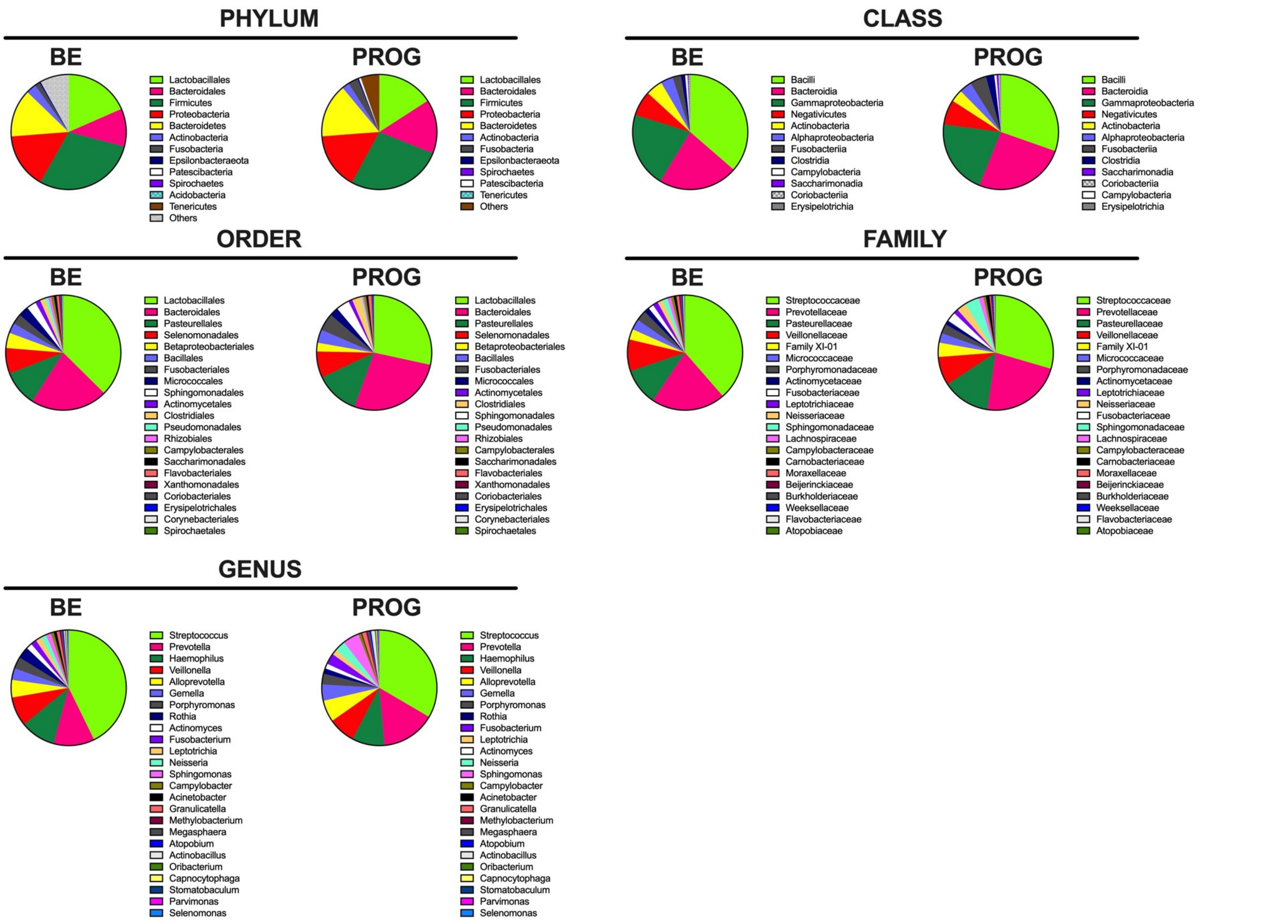
PHYLUM
BE
PROG
Lactobacillales
Bacteroidales
Firmicutes
Proteobacteria
Bacteroidetes
Actinobacteria
Fusobacteria
Epsilonbacteraeota
Patescibacteria
Spirochaetes
Acidobacteria
Tenericutes
Others
Lactobacillales
Bacteroidales
Firmicutes
Proteobacteria
Bacteroidetes
Actinobacteria
Fusobacteria
Epsilonbacteraeota
Spirochaetes
Patescibacteria
Tenericutes
Others
CLASS
BE
PROG
Bacilli
Bacteroidia
Gammaproteobacteria
Negativicutes
Actinobacteria
Alphaproteobacteria
Fusobacteriia
Clostridia
Campylobacteria
Saccharimonadia
Coriobacteriia
Erysipelotrichia
Bacilli
Bacteroidia
Gammaproteobacteria
Negativicutes
Actinobacteria
Alphaproteobacteria
Fusobacteriia
Clostridia
Saccharimonadia
Coriobacteriia
Campylobacteria
Erysipelotrichia
ORDER
BE
PROG
Lactobacillales
Bacteroidales
Pasteurellales
Selenomonadales
Betaproteobacteriales
Bacillales
Fusobacteriales
Micrococcales
Sphingomonadales
Actinomycetales
Clostridiales
Pseudomonadales
Rhizobiales
Campylobacterales
Saccharimonadales
Flavobacteriales
Xanthomonadales
Coriobacteriales
Erysipelotrichales
Corynebacteriales
Spirochaetales
Lactobacillales
Bacteroidales
Pasteurellales
Selenomonadales
Betaproteobacteriales
Bacillales
Fusobacteriales
Micrococcales
Actinomycetales
Clostridiales
Sphingomonadales
Pseudomonadales
Rhizobiales
Campylobacterales
Saccharimonadales
Flavobacteriales
Xanthomonadales
Coriobacteriales
Erysipelotrichales
Corynebacteriales
Spirochaetales
FAMILY
BE
PROG
Streptococcaceae
Prevotellaceae
Pasteurellaceae
Veillonellaceae
Family XI-01
Micrococcaceae
Porphyromonadaceae
Actinomycetaceae
Fusobacteriaceae
Leptotrichiaceae
Neisseriaceae
Sphingomonadaceae
Lachnospiraceae
Campylobacteraceae
Carnobacteriaceae
Moraxellaceae
Beijerinckiaceae
Burkholderiaceae
Weeksellaceae
Flavobacteriaceae
Atopobiaceae
Streptococcaceae
Prevotellaceae
Pasteurellaceae
Veillonellaceae
Family XI-01
Micrococcaceae
Porphyromonadaceae
Actinomycetaceae
Leptotrichiaceae
Neisseriaceae
Fusobacteriaceae
Sphingomonadaceae
Lachnospiraceae
Campylobacteraceae
Carnobacteriaceae
Moraxellaceae
Beijerinckiaceae
Burkholderiaceae
Weeksellaceae
Flavobacteriaceae
Atopobiaceae
GENUS
BE
PROG
Streptococcus
Prevotella
Haemophilus
Veillonella
Alloprevotella
Gemella
Porphyromonas
Rothia
Actinomyces
Fusobacterium
Leptotrichia
Neisseria
Sphingomonas
Campylobacter
Acinetobacter
Granulicatella
Methylobacterium
Megasphaera
Atopobium
Actinobacillus
Oribacterium
Capnocytophaga
Stomatobaculum
Parvimonas
Selenomonas
Streptococcus
Prevotella
Haemophilus
Veillonella
Alloprevotella
Gemella
Porphyromonas
Rothia
Fusobacterium
Leptotrichia
Actinomyces
Neisseria
Sphingomonas
Campylobacter
Acinetobacter
Granulicatella
Methylobacterium
Megasphaera
Atopobium
Actinobacillus
Oribacterium
Capnocytophaga
Stomatobaculum
Parvimonas
Selenomonas

Figure S1. Pie charts representing the difference in the relative abundance of the main bacteria at each taxonomic level, colonizing the esophageal tissue comparing non-dysplastic BE patients and patients who progressed to LGD, HGD, and EAC identified as “PROG”. Data are presented as median values. Only data showing a median value of relative abundance higher than 0.01% were reported.
